# Maslinic acid promotes autophagy by disrupting the interaction between Bcl2 and Beclin1 in rat pheochromocytoma PC12 cells

**DOI:** 10.18632/oncotarget.20210

**Published:** 2017-08-05

**Authors:** Xiaoli Dong, Jiaxiao Zhang, Zhilin Zhou, Zhennan Ye, Jiahao Chen, Jifan Yuan, Fengjun Cao, Xuanbin Wang, Wenchao Liu, Wenxuan Yu, Xiaohua Li

**Affiliations:** ^1^ The Hong Kong Polytechnic University Shenzhen Research Institute, Shenzhen, PRC; ^2^ Department of Research and Development, Shenzhen Benevop Biomedical Co., Ltd, Shenzhen, PRC; ^3^ Department of General Surgery, Liyuan Hospital, Tongji Medical College, Huazhong University of Science and Technology, Wuhan, PRC; ^4^ Department of Biochemistry II, Jena University Hospital, Jena, Germany; ^5^ Laboratory of Chinese Herbal Pharmacology, Oncology Center, Renmin Hospital, Hubei University of Medicine, Shiyan, PRC; ^6^ Hubei Key Laboratory of Wudang Local Chinese Medicine Research, Shiyan, PRC; ^7^ Department of Applied Biology and Chemical Technology, The Hong Kong Polytechnic University, Hong Kong, PRC; ^8^ State Key Laboratory of Chinese Medicine and Molecular Pharmacology (Incubation), Shenzhen, PRC

**Keywords:** maslinic acid, autophagy, regulation, Beclin1, Bcl2

## Abstract

Maslinic acid (2α, 3β-dihydroxyolean-12-en-28-oic acid, MA) was isolated from natural plants and showed anti-cancer activity in rat Pheochromocytoma PC12 cells in our previous studies. We now discover that MA disrupts the interaction between Bcl2 and autophagy scaffold protein Beclin1 in the above cell line, leading to the up-regulation of autophagy. We investigated the effect of MA on the interaction between Bcl2 and Beclin1 by biochemical and biophysical methods in combination with autophagy characterization in the above cell line. Our results suggest that MA may serve as an autophagy activator by directly blocking the Bcl2-Beclin1 interaction to release free Beclin1 required for the recruitment of autophagy positive regulators, implying MA may exert its anti-cancer activity by regulating autophagy.

## INTRODUCTION

**Figure 1:**
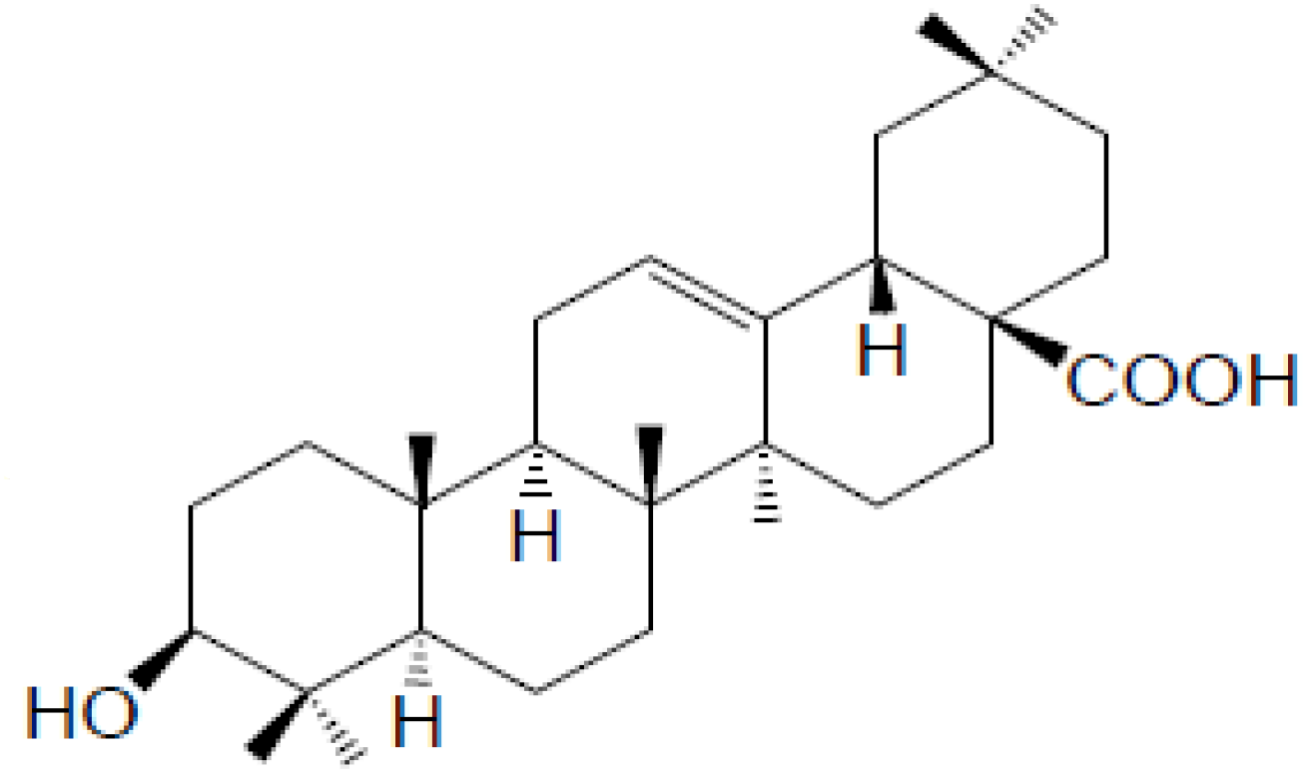
Chemical structure of maslinic acid (MA)

Maslinic acid, abbreviated from 2α, 3β-dihydroxyolean-12-en-28-oic acid, MA, a member of triterpenes (Figure 1), is widely found in nature and is mainly present in olive-pomace oil (an olive skin wax), being the principal component of the wax-like coating in the olive skin [1]. MA was reported to exhibit anti-tumor [2], antioxidant activities [3], have antiallodynic and analgesic effects [4], and anti-inflammation [5], anti-virus [6], parasitostatic effects [7].

MA has been shown to possess anti-tumor effects on several types of tumor cells, including colon [8, 9], ovary and melanoma [10], cardiac carcinoma [11], bladder cancer [12], breast cancer [13], pancreatic cancer, non-small cell lung cancer as well as tumor cellsin the central nervous system [8]. Apoptosis is involved in the mechanism of action by which MA suppresses the viability of a wide range of cancers [12] by regulating many downstream signaling pathways that are activated through PKC receptors [14]. The involved pathways include the p38 MAPK/mitochondria/caspase pathway [11, 12] and NF kappa B signaling pathway [2]. However, the exact working target of MA in cancer cells requires further investigation.

In addition to apoptosis, autophagy is closely related to cancer therapy. Autophagy is the process by which the unwanted cellular components are enwrapped by double-membrane structured vesicles that will eventually be fused with the lysosome for degradation and reuse [15]. Autophagy and apoptosis share similar regulation pathways involving the same initiator or effector molecules, and even engage common cellular organelles and/or sites to exert their functions [16]. However, autophagic cell death is caspase-independent and involves an increased formation of double-membrane structured vesicle known as autophagosome which is the essential characteristic of autophagy [17]. Furthermore, most of the proteins that participate in the regulation of autophagy are either tumor suppressors or oncogenic factors; mechanism underlying the regulation of autophagy largely overlaps with signaling pathway involved in tumorigenesis [18]. Autophagy is involved in different phases of cancer development, impeding early cancer development while facilitating advanced tumor progression [19].

**Figure 2:**
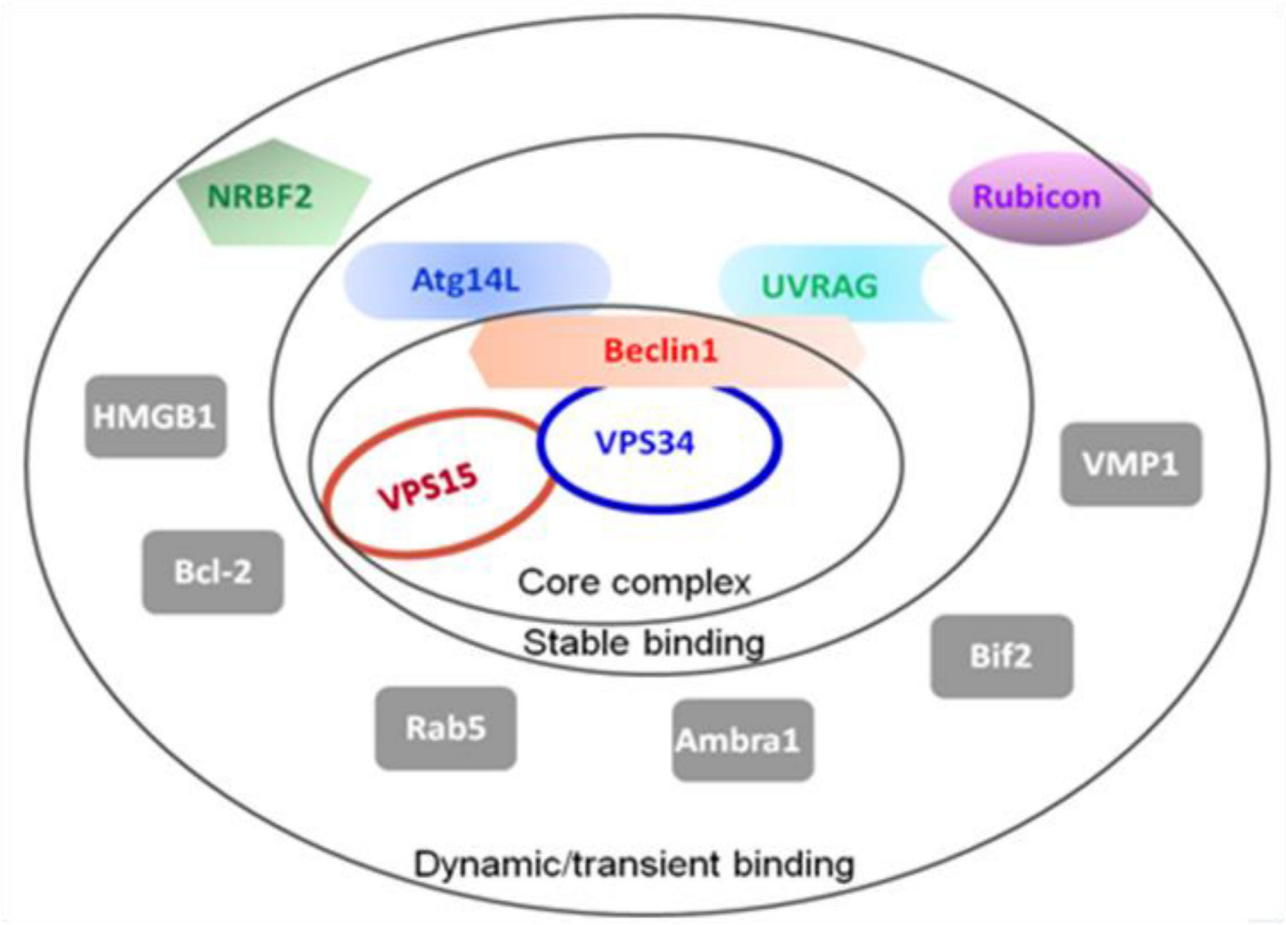
Summary of the autophagy-related proteins (ATGs) The Beclin1-VPS34-VPS15 serves as the core complex, recruiting other ATGs with different binding affinities to regulate the VPS34 kinase activity.

There are three types of autophagy, macroautophagy, microautophagy and chaperone-mediated autophagy, which differ with respect to their function and the mode of delivery of cargo to the lysosomes [20]. Macroautophagy is the major lysosomal degradation pathway involving the formation of autophagosomes, in which over 30 autophagy-related genes (ATGs) participate as summarized in Figure 2 [21].

Autophagosome formation and maturation is a multi-step process highly regulated and controlled by ATGs. Initiation phase of autophagosome formation is controlled by the ULK and class III PI3K complex [22]. ULK complex is regulated by upstream kinases or autophagy-related factors according to cellular nutrient and energy status, resulting in phosphorylation on ULK 1/2 proteins for inhibition or stimulation. The Class III PI3K complex, after activated by ULK-dependent phosphorylation, is in charge of the production of PI3P, which is required for initiation of the isolated double-membrane structure called autophagophore (precursor of autophagosome) [22]. Subsequently, PI3P-binding proteins and proteins in ubiquitin-like conjugation system are recruited to elongate and expand the autophagophore, resulting in the maturation of autophagosome, which is closed and surrounds the unwanted proteins or organelles for degradation [20–22].

Autophagy can result in contradictory outcomes in cancer cells, survival or death [23]. Anticancer agents that induce autophagy could drive cancer cells into non-apoptotic programmed cell death when apoptosis is defective. Conversely, inhibition of autophagy prevents cancer cellsfrom using autophagy as a survival strategy during therapy [17]. Drugs targeting the negative autophagic regulators including Bcl2 or epidermal growth factor receptor (EGFR) have the capacity to induce autophagy, thus they should be used modestly in anticancer therapy [24].

Beclin1, as a scaffold protein in the Class III PI3K complex, is an interaction platform to recruit other ATGs for autophagy regulation [25]. Beclin1 interacts with Bcl2 via its BH3 domain, leading to down-regulation of autophagy by inhibiting the formation and activation of the Class III PI3K complex [26, 27]. Besides the insertion and conversion of LC3/Atg8 on autophagophosomal membrane, Beclin1-including Class III PI3K complex is essential for the generation and maturation of autophagosomes [28, 29]. LC3/Atg8, as an ubiquitin-like protein, has two forms named LC3-I and LC3-II, while the latter is widely used to characterize the level of autophagy for it consistently accumulates during the autophagosomal formation [14, 28, 29]. Antibodies recognizing LC3 are commonly used to generate the signal of punctate for autophagosome in the immunohistochemistry and immunocytochemistry characterization [20].

Activation of the Class III PI3K complex is incompatible with interaction between Bcl2 and Beclin1 [26]. In the present study, MA was proved to induce cell autophagy by disrupting the combination of Bcl2 and Beclin1 in rat PC12 cells, suggesting a novel molecular target of MA in cancer therapy.

## RESULTS

### MA treatment promoted LC3-I/II conversion in rat pheochromocytoma PC12 cells

**Figure 3:**
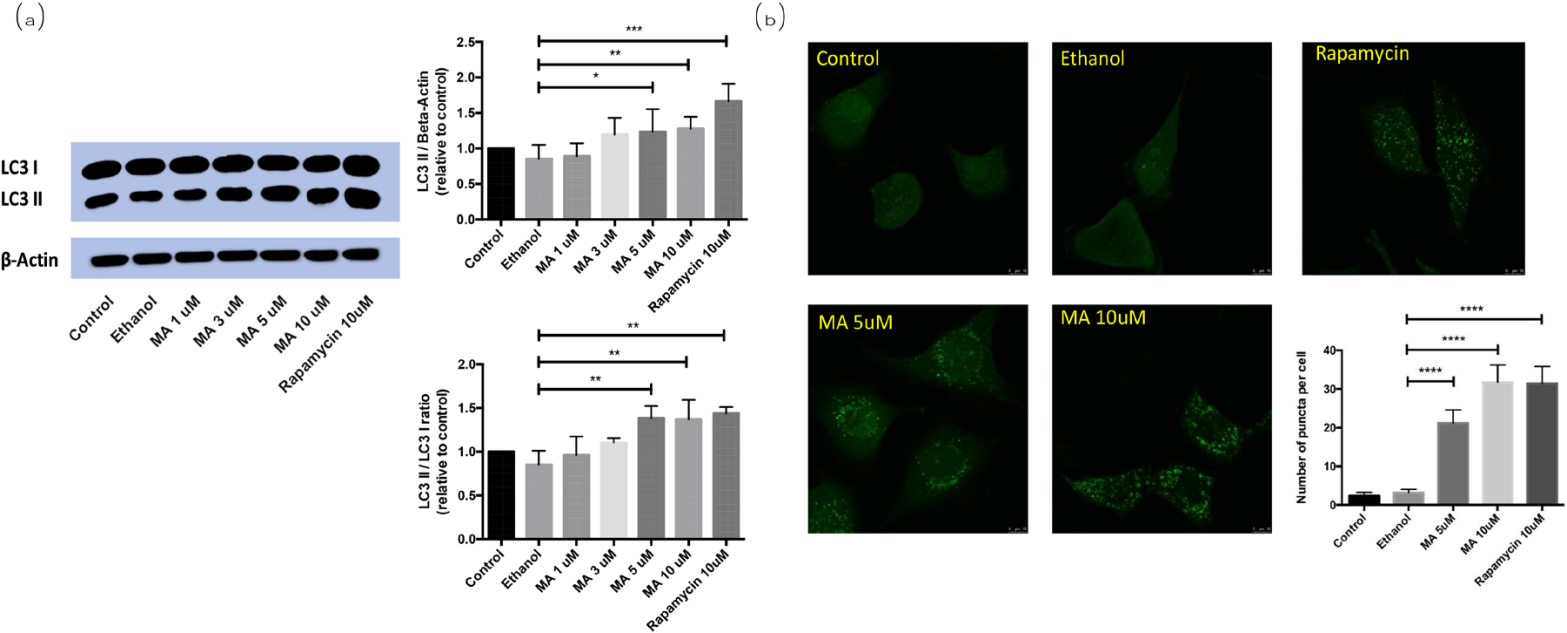
Effect of MA on LC3-I/II conversion in PC12 cells MA treatment released Beclin1 and promoted autophagy in rat pheochromocytoma PC12 cells. **(a)** Western blot analysis; **(b)** immunofluorescence analysis.

Western blot analysis of the transformation from LC3-I to LC3-II was performed on the rat pheochromocytoma PC12 cells. With the dosage of MA increased from 1 μM to 10 μM, the endogenous level of LC3-II was significantly increased accordingly (Figure 3a, 3b). The immunofluorescence capture for LC3 puncta, the marker of autophagosome, also showed MA can promote the formation of autophagosomes, implying it can positively regulate autophagy.

### The interaction between Beclin1 and Bcl2 was inhibited by the presence of MA

**Figure 4:**
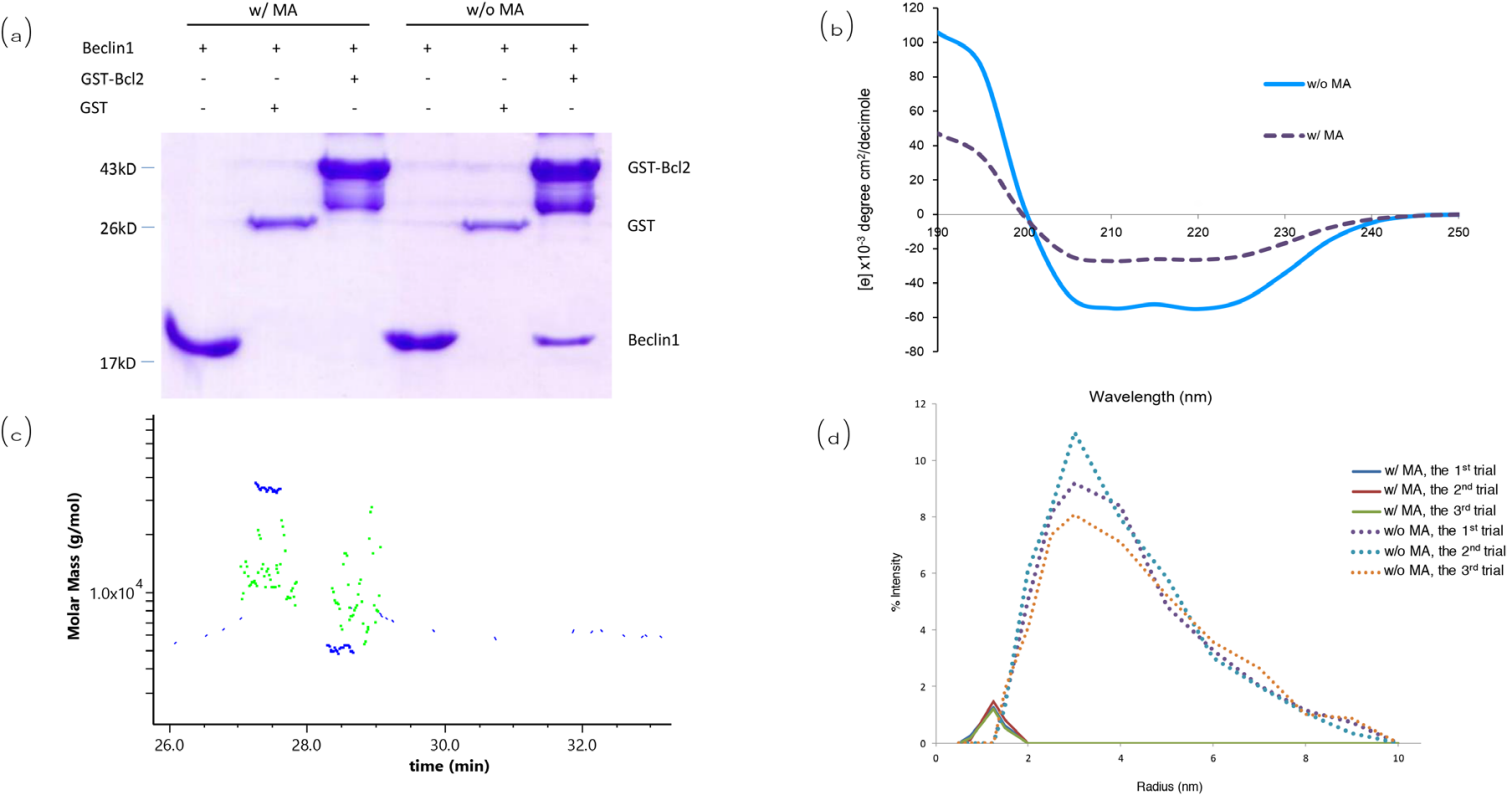
Analysis for the interaction between Beclin1 and Bcl2 **(a)** Pull-down assay. **(b)** Circular dichroism. **(c)** Detection of apparent molecular weight for Beclin1-Bcl2 by FPLC-LS. When MA not included (blue), the molecular weight detected is about 35 kD, matching the theoretical molecular weight of Beclin1-Bcl2 complex; when MA included (green), the molecular weight detected is in the range of 8-20 kD, indicating Beclin1 and Bcl2 are monomers. **(d)** Determination of protein size by dynamic light scattering. The window within 1-12 nm (usually protein particle size range) displays the spectrum shifted left when MA included, meaning the size of protein particle has become smaller (interaction between Beclin1 and Bcl2 destroyed by MA).

Pull-down assay showed that Beclin1 was pulled down by Bcl2 in the absence of MA (lane 6, Figure 4a), while it wasn’t pulled down by Bcl2 in the presence of MA (lane 3, Figure 4a). The CD spectrum of the coiled structure at 20°C showed a pattern with a double bottom at wavelengths 208 and 222 nm, which is characteristic of the coiled coil structural motif. The presence of MA led to a gradual loss of the double-bottom feature at 208 and 222 nm, implying the coiled structure of Beclin1-Bcl2 complex was significantly reduced (Figure 4b). The data implied that the interaction between Beclin1 and Bcl2 could be blocked by MA. Besides, detection of molecular weight for Beclin1-Bcl2 mixture (molar ratio 1:1) by FPLC-LS showed the molecular weight detected was about 35 KD when MA was not included in the buffer (Figure 4c), which matches the theoretical molecular weight of Beclin1-Bcl2 complex; while the molecular weight detected was in the range of 8-20 KD when MA was included in the buffer (Figure 4c), which matches the molecular weight of Beclin1 or Bcl2 monomer, indicating Beclin1- Bcl2 interaction was blocked. Therefore, the size distribution of Bcl2-Beclin1 complex experienced a conformational change from dimer to monomers in Tris-HCl buffer solution (pH = 7.4) after the addition of MA. Afterwards, determination of protein size was conducted for the Beclin1-Bcl2 mixture by dynamic light scattering. The diameter of aggregates that were composed of Bcl2-Beclin1 complex was around 3.4 nm; it was decreased to around 1.6 nm in the presence of MA (Figure 4d), consistent with the result of measurement for Beclin1 or Bcl2 alone. The window within 1-12 nm (usually protein particle size range) displayed a left-shifted spectrum when MA was included, meaning the size of protein particle had become smaller, indicating the disruption of interaction between Beclin1 and Bcl2 in the presence of MA (Figure 4d).

### MA directly blocked the interaction between Beclin1 and Bcl2

**Figure 5:**
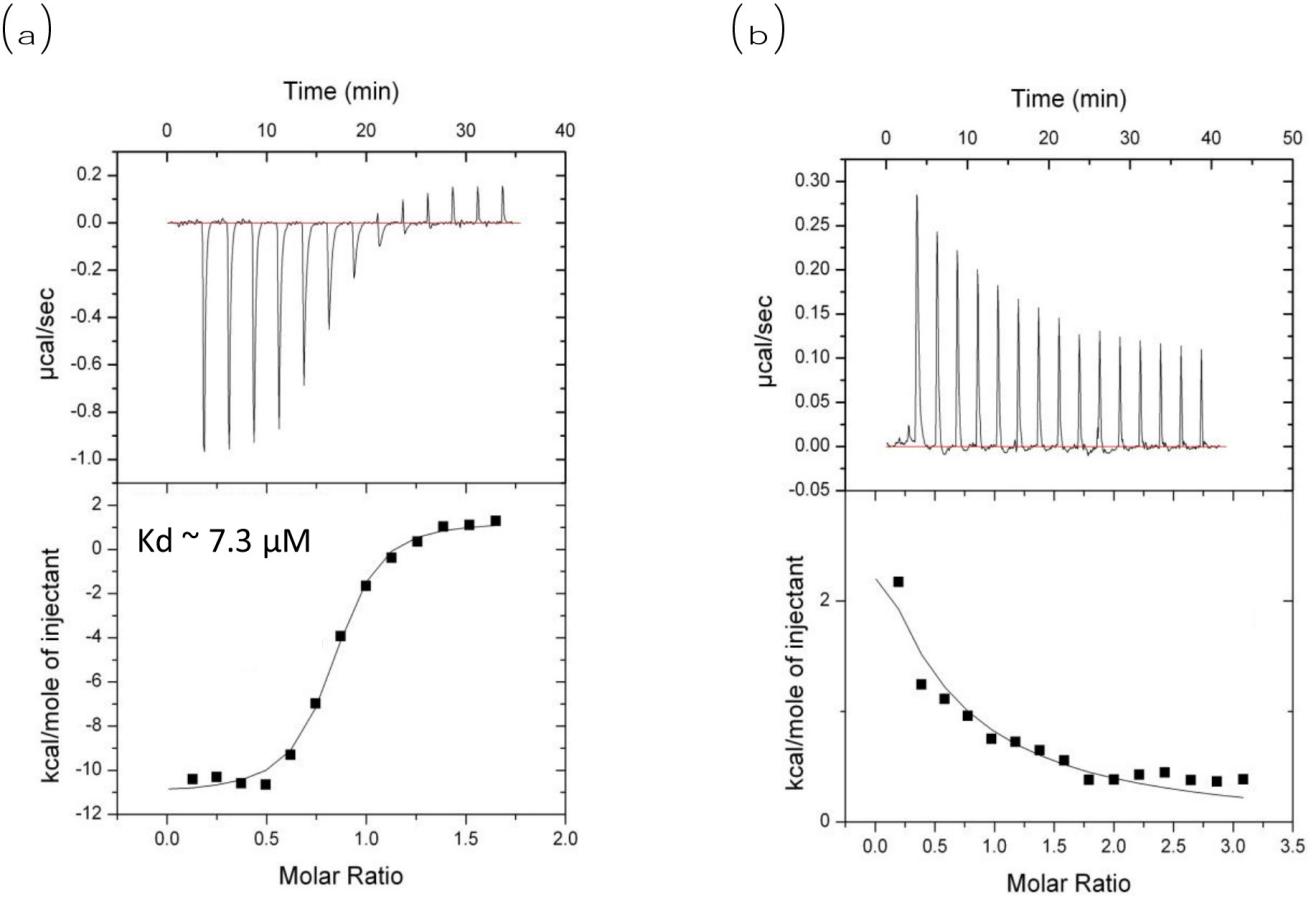
ITC measurement of Bcl2 and Beclin1 interaction in the absence or presence of MA ITC measurement for interaction between Beclin1 and Bcl2. **(a)** No MA included, exothermic reaction observed when Beclin1 titrated into Bcl2 (negative ordinate indicates exothermic); **(b)** MA included, endothermic reaction observed when Beclin1 titrated into Bcl2 (positive ordinate indicates endothermic).

The effect of MA on the interaction between Beclin1 and Bcl2 was quantified by ITC measurements (Figure 5). Our ITC data showed an exothermic reaction with the kd of ∼7.3 μM when Beclin1 was titrated into Bcl2 (negative ordinate indicates exothermic reaction) (Figure 5a); when 10 μM of MA was included in the Tris-HCl buffer, endothermic reaction was observed upon Beclin1’s titration into Bcl2 (positive ordinate indicates endothermic reaction), indicating a heat transfer due to dilution (Figure 5b).

### MA treatment decreased the co-localization of Beclin1 and Bcl2

**Figure 6:**
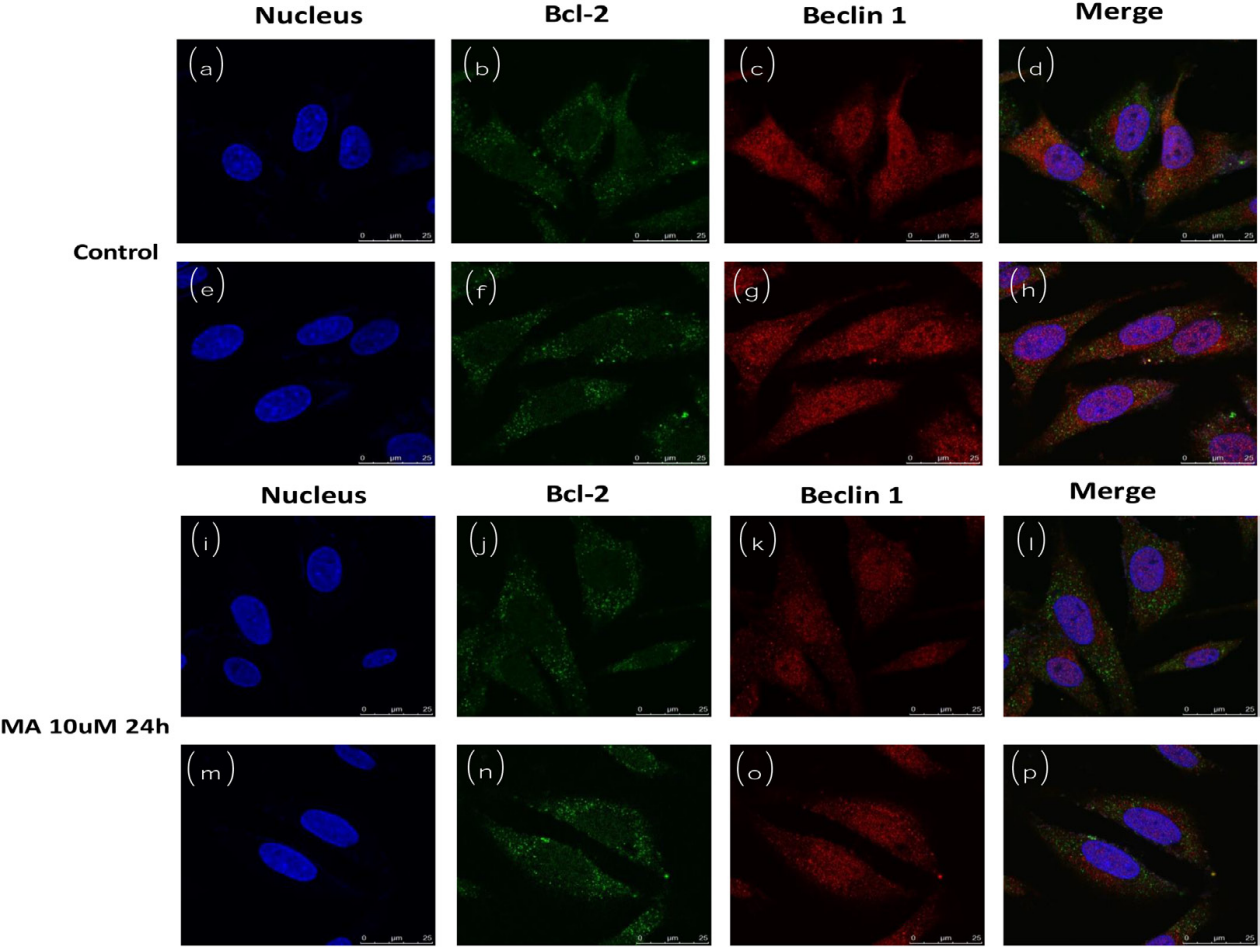
Cofocal imaging for autophagosome (subcellular localization of Beclin1 and Bcl2) in the absence or presence of MA

Endogenous Beclin1 and Bcl2 were co-localized in the cytoplasm (Figure 6d, 6h). However, the co-localization was dramatically decreased when MA was added to the medium (Figure 6l, 6p), implying the Beclin-Bcl2 complex was dissociated, releasing either Beclin1 or Bcl2 free. The control was solvent without MA.

### The co-immunoprecipitation of Beclin1 by over-expressed Bcl2 was interrupted by the presence of MA

**Figure 7:**
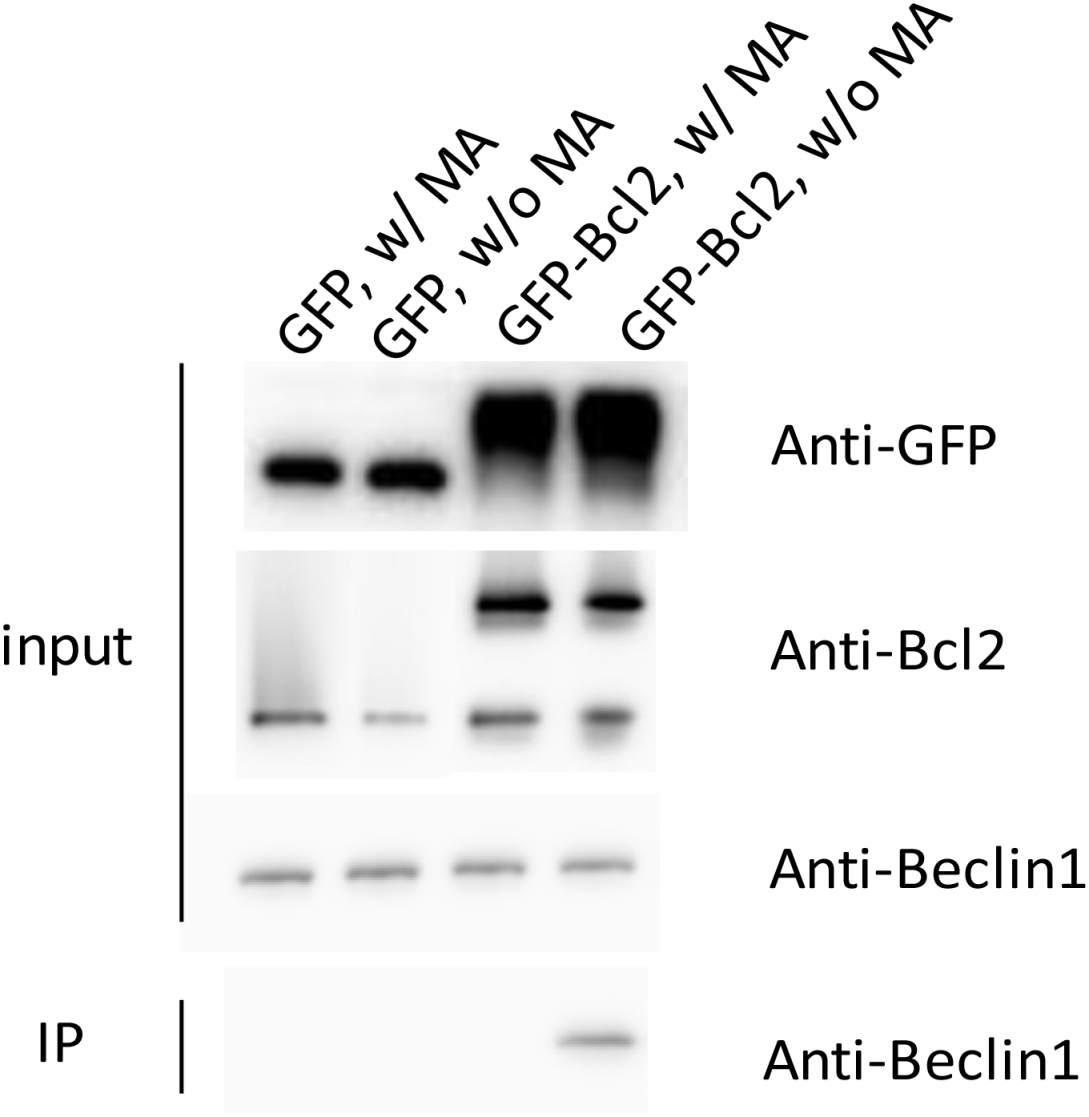
The co-immunoprecipitation of Beclin1 by overexpressed Bcl2 was interrupted by the presence of MA

We overexpressed GFP-Bcl2 in rat pheochromocytoma PC12 cells to conduct co-IP experiments to demonstrate the interaction between Bcl2 and endogenous Beclin1 (for details please refer to the section on methods) for the expression level of endogenous Bcl2 was not sufficient for the detection in western blot analysis (data not shown). The data showed endogenous Beclin1 could be co-immunoprecipitated by overexpressed Bcl2, while such result was not seen in the presence of MA, implying that the Bcl2-Beclin1 interaction was negatively affected by MA (Figure 7).

### Molecular docking suggested MA competes with Bcl2 for Beclin1 binding

Zhang’s group has suggested Beclin1 BH3 directly interacts with Bcl2 [27]. Our current study showed MA is capable of blocking the Bcl2-Beclin1 interaction. In order to understand the mechanism, molecular docking of MA with Bcl2/Beclin1 was done, which showed a direct binding of MA to the pocket of Beclin1 BH3 domain through the hydrogen bonds formed between carboxyl group oxygen and the nitrogen in the side chain of ARG87 in Beclin1, associated with the hydrophobic interactions with the surrounding including Leu74, Tyr60, Leu78 and Phe48. The interaction sites are overlapped with those of Bcl2-Beclin1 interaction, explaining why MA can negatively affect their binding.

**Figure 8:**
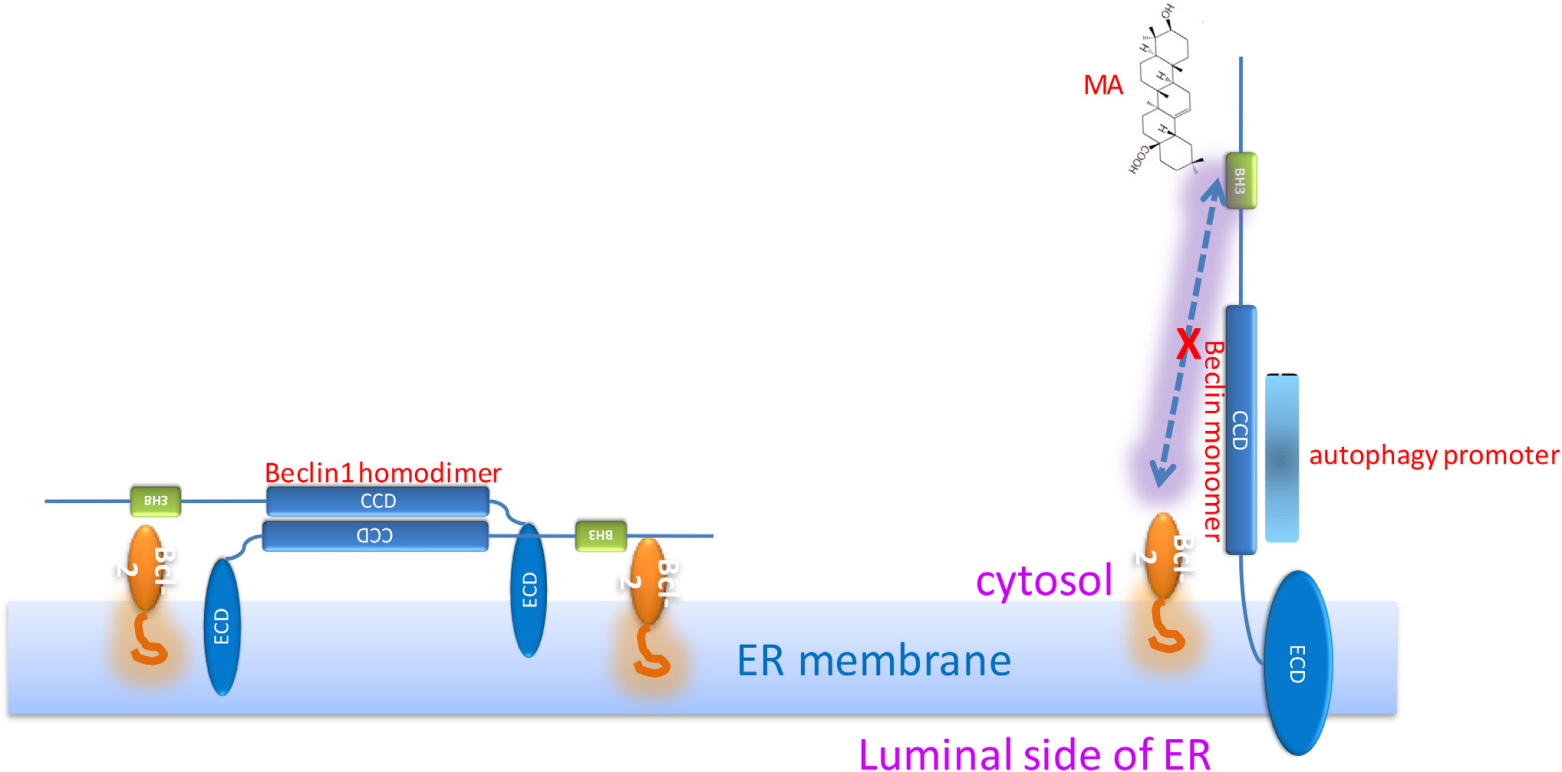
Hypothesis: Bcl2-Beclin1 interaction strengthens Beclin1 homodimer; MA-Beclin1 interaction leads to the Beclin1 monomer required for the recruitment of autophagy promoter(s)

Beclin1 contains three functional domains: the evolutionarily conserved domain (ECD) for anchoring on the membranes including ER membrane, the coiled-coil domain (CCD) for recruitment of autophagy regulators, and the BH3 domain which is capable of binding to Bcl2. Our previous studies have solved the crystal structure of Beclin1 CCD to find it is an antiparallel dimer and demonstrated that this structural feather allows Beclin1 to serve as a reserve pool ready for autophagy promoters including Atg14 and UVRAG [25]. All the available information together promotes the binding pattern as shown in Figure 8. When Bcl2 binds to the Beclin1 BH3 domain, Beclin1 is forced to stay in homodimer, meaning the interaction sites on Beclin1 CCD for autophagy promoters are not accessible. However, in the presence of MA, Beclin1 is subsequently released from the restriction of Bcl2 binding and stays in monomer to expose the interaction sites on Beclin1 CCD for autophagy promoters binding, leading to the assembly and activation of PI3K complex (Figure 2), which is crucial for autophagosome formation which in turn would result in the upregulation of autophagy.

**Figure 9:**
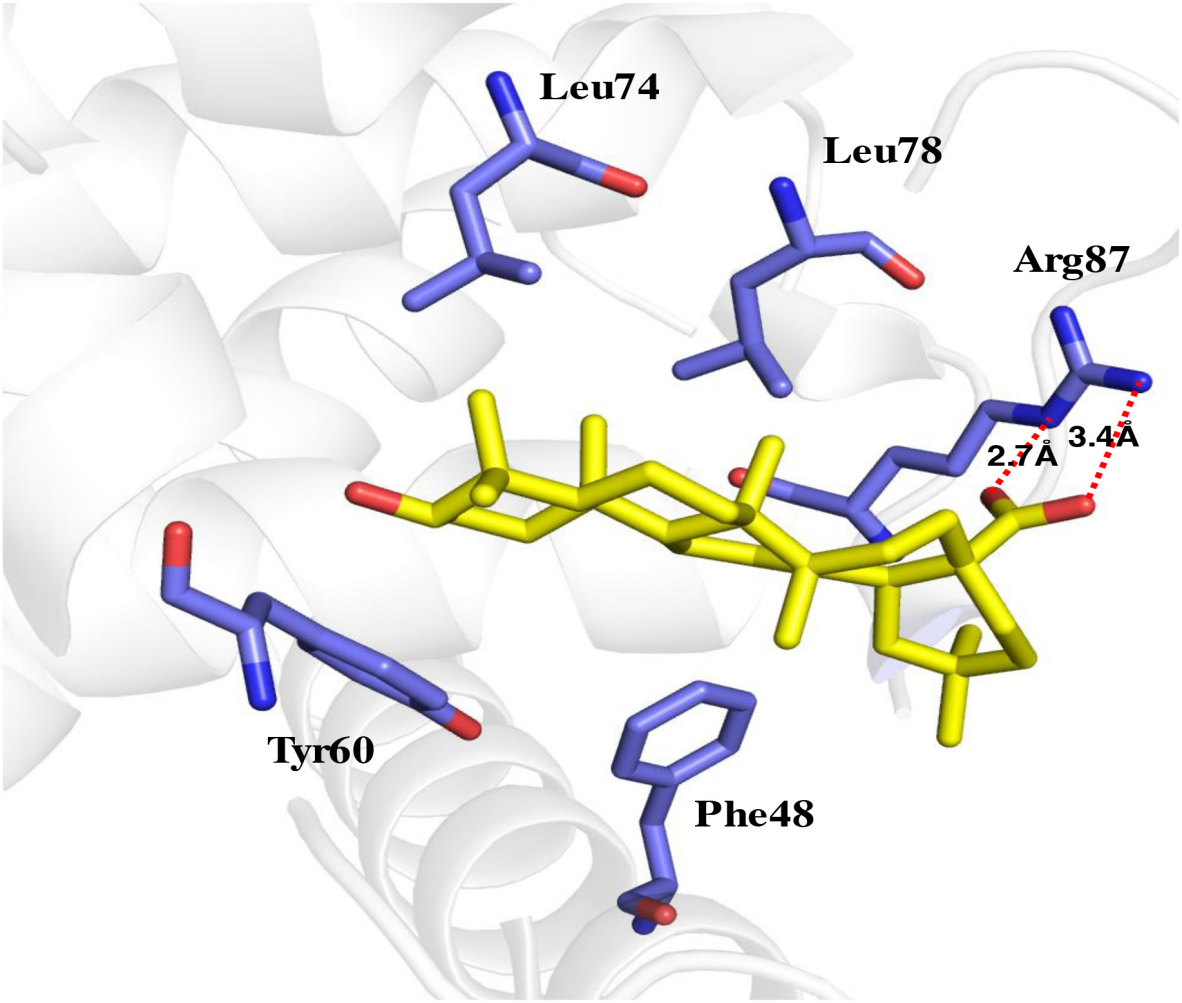
Molecular docking of MA binding to Beclin1 BH3 domains

We subsequently proposed a hypothesis to better understand our results: MA competes with Bcl2 for the binding of the Beclin1 BH3 domain, thus releasing free Beclin1 for autophagy promoters. Beclin1 is a scaffold protein in autophagy to recruit other autophagy regulators (Figure 2) including Atg14L and UVRAG through its coiled-coil domain. Simulation by molecular docking was carried out for confirmation of this hypothesis. It showed MA could bind to the BH3 domain of Beclin1 (Figure 9), resulting in the release of free Beclin1 for autophagy promoters. We further measured the binding affinity of MA and Beclin1 by ITC measurement and found the binding constant was not as strong as in a significant binding (data not shown). Therefore, the overall effect of MA is probably to disrupt the Bcl2-Beclin1 interaction and release Beclin1 for Atg14L and/or other positive regulators’ access in autophagy (Figure 8).

## DISCUSSION

Previous studies on MA have suggested that it possesses pharmacological functions including anti-inflammation, anti-oxidation and anti-tumor. This anti-tumor function, which has attracted the most attention [30–33], is reported to be closely related to apoptotic pathway [12, 34–36] but the underlying mechanism remains to be elucidated. One study investigated the expression level of several inflammatory factors, allowing the authors to argue that MA exerts anti-tumor effect by preventing chronic inflammation which represents a crucial step in the development of some cancers [37]. However, the evidence of molecular mechanism is to be provided.

Autophagy is a fundamental metabolic cellular process for the turnover of unwanted proteins or organelles to recycle nutrients for cell survival. Autophagy is highly concerned in anti-cancer studies although its functions in cancers have not been clearly studied so far [38, 39]. Notably, there is a growing number of natural molecules reported to both exert anti-tumor affects and play roles in autophagy regulation [40]. However, whether the anti-tumor effect is achieved through autophagy remains unclear.

MA showed better antioxidant activity under stressful condition [41], and anti-neurodegeneration activity in PC12 cells (differentiated by nerve growth factor against beta-amyloid-induced apoptosis) [42]. It is well known that the Bcl2-Beclin1 interaction is the mechanism regulating autophagy/apoptosis toggle switch, serving as the platform of cross-talk in apoptosis and autophagy [43]. Bcl2 binds strongly to Beclin1 both *in vitro* and *in vivo* [27]. Our studies showed the Bcl2-Beclin1 interaction was blocked in the presence of MA, suggesting that the anti-tumor effect of MA probably be exerted through autophagy regulation. Our study reported the molecular mechanism in the biological activities of MA for the first time, providing clue for the researches focused on the development of MA derivatives, aiming to improve the bioactivities of MA [44, 45].

## MATERIALS AND METHODS

### Reagents

Maslinic acid (2α, 3β-dihydroxyolean-12-en-28-oic acid, MA) was purchased from Sigma-Aldrich (St.Louis, MO, USA). The purity of MA is ≥98% (HPLC). MA was fully dissolved in 100% ethanol before use. Anti-LC3, anti-p62, anti-Bcl2, anti-Beclin1 antibodies were purchased from Cell Signaling Technology (Danvers, MA, USA). HRP-goat anti-rabbit conjugate and HRP-goat anti-mouse conjugate were purchased from Santa Cruz Technology Inc (Santa Cruz, CA, USA). Glutathione Sepharose was purchased from GE Healthcare (Chicago, IL, USA). Alexa Fluor 488 conjugate and Alexa Fluor 594 conjugate, cell culture media and serum were purchased from Invitrogen (Waltham, MA, USA). Other chemicals and reagents were purchased from Sigma-Aldrich (St. Louis, MO, USA).

### Cell culture

Rat adrenal pheochromocytoma (PC12) cells were purchased from ATCC (American Tissue Culture Collection, Rockville, MD) and cultured in a humidified incubator at 37°C with 5% CO_2_. PC12 cells were maintained in Dulbecco’s Modified Eagle Medium (DMEM) supplemented with 10% fetal bovine serum (FBS), 1% horse serum (HS) and 1% penicillin/streptomycin. Before drug treatment, the cells were sub-cultured in a 6-well plate and the medium was changed to DMEM containing 2% FBS and 1% HS. The next day after cell adhesion, different concentrations of maslinic acid (MA) and the positive control of Rapamycin (Rap) (10 μM) were be added to different wells and incubated for 24 hours. Then cells were washed by PBS and applied for western blot analysis or fluorescent microscope imaging. All cells were passaged fewer than 6 months after resuscitation and cultured using the protocol provided by ATCC.

### Protein expression and purification

The BH3 domain and Coiled Coil domain (Beclin1 hereafter) of rat Beclin1 (residues 100–266) were cloned as a 6*His-tagged protein in a modified pET32 vector containing the human rhinovirus 3C protease cleavage site and without thioredoxin fusion. The BH3 motif containing region of rat Bcl2 (residues 1–156) was cloned as GST-fusion protein into a modified pET32 vector (Novagen) containing the 3C protease cleavage site. Both constructs were expressed at 30°C in *E. coli* BL21 (DE3) cells after isopropyl-β-d-thiogalactopyranoside induction and purified by affinity chromatography (HisTrap HP, GE Healthcare). Further purification after 3C cleavage was carried out using size-exclusion chromatography (Superdex 75, GE Healthcare). For pull-down assay, the GST-tagged version of Bcl2 was expressed under the same condition as the un-tagged version, and purified by affinity column chromatography with no protease cleavage.

### Pull-down assay

Pull-down assay was used to detect the interaction between Beclin1 and Bcl2. The purified GST or GST-tagged Bcl2 was the bait, and the purified autophagy scaffold protein Beclin1 was the prey. They were mixed together in a molar ratio of 1:1 in a buffer containing 50 mM Tris, 150 mM NaCl, pH 8.0 in the absence or presence of MA. Two concentrations of MA, 20 μM and 10 μM, were tested in our experiments. The mixture was incubated at room temperature for 30 minutes before it was added to the GST affinity agarose beads equilibrated by the above buffer in advance. After the standard procedures of pull-down assay, the protein complex was eluted out and loaded on SDS-PAGE for separation and detection.

### Circular dichroism spectroscopy

CD full-length scanning spectra were collected between 190 and 250 nm at 20 °C using a quartz cell with a 0.1 cm path length on a Jasco J-810 spectropolarimeter equipped with thermoelectric temperature control. Measurements were made on protein samples at 50 μM concentration in 50 mM NaCl, 20 mM Tris buffer, pH 8.0. Spectra were collected at 1.0 nm intervals with a 5-s averaging time per data point. A reference spectrum generated from a scan of the buffer without sample was subtracted before data were converted to mean residue ellipticity.

### Light scattering

FPLC-LS were used to measure the molecular weight of Beclin1 and Bcl2. Dynamic light scattering (DLS) was used to measure the size and diameter of protein structure. 1.0 mM of the purified Bcl2 was mixed with 1.0 mM of purified Beclin1 (molar ratio 1:1) in a buffer containing 50 mM Tris, 150 mM NaCl, pH 8.0 in the presence or absence of a final concentration of 10 μM MA. The mixture was incubated for 30 minutes at room temperature before it was loaded to size exclusion chromatography (Superdex 75, GE Healthcare) by HPLC. The retention time and molecular weight of each separated fraction were recorded by static light scattering instrument, while the size (diameter) of protein was measured by dynamic light scattering instrument. Raw data were presented here.

### Isothermal titration calorimetry (ITC) measurements

Isothermal Titration Calorimetry was performed using Auto-iTC200 (GE, USA). Samples were dialysed into 50 mM Tris, pH 8.0, and 150 mM NaCl before experiment. To study the interaction between Beclin1 and Bcl2 in the absence of MA, we used a typical titration consisting of injecting 2 μl aliquots of 200 μM Beclin1 solution into 200 μl aliquots of 20 μM Bcl2 solution after every 1 min to ensure the titration peak would return to the baseline prior to the next injection. Aliquots of the same Beclin1 solutions were injected into only the reaction buffer (50 mM Tris containing 150 mM NaCl, pH 8.0) in separate ITC runs to measure the heats of dilution of the ligands. To study the interaction between Beclin1 and Bcl2 in the presence of MA, 200 μM Beclin1 solution was titrated into 200 μl aliquots of 20 μM Bcl2 solution containing 10 μM MA with the settings as above. Control experiments were performed by titrating Beclin1 solution into the same buffer to obtain the heats of dilution. Typically, titrations consisted of 20 injections of 2 μl, with 180-s equilibration between injections. Each reaction was repeated 3 times. The data were analysed using Origin 7.0.

### Western blot analysis

Cell proteins were obtained by cell lysis buffer with protease inhibitors (Cell signaling, MA, USA). Protein concentrations were measured by Bradford protein assay (Bio-Rad Laboratory, USA). Equal amounts of cytosolic proteins (15 μg) were mixed with the loading dye. After boiling, the samples were separated by SDS-PAGE and transblotted to PVDF membranes (Immobilin-P, Millipore Corp., Bedford, MA, USA). The blots were probed first with polyclonal rabbit anti-human LC3 (Cell signaling Technology, Beverly, MA, USA), followed by incubation with goat anti-rabbit IgG/horseradish peroxidase (Cell signaling Technology, Beverly, MA, USA). The antigen-antibody complexes were detected by using an enhanced chemiluminescence reagent and visualized by a Lumi-Imager with the software. The level of β-actin was also detected and used as an internal control for equal loading of protein samples.

### Fluorescent microscopy imaging

After treatment, cells were fixed in fresh 4% paraformaldehyde for 20 min, and permeablized with 0.1 % Triton X-100 in PBS for 5 min at room temperature. After blocking in PBS containing 5 % BSA for 30 min, cells were incubated in primary antibodies (1:100) (anti-Bcl2, anti-Beclin1) overnight at 4°C. After three washes in PBS, cells were incubated with fluorescent secondary antibodies (Alexa Fluor 488 conjugate anti rabbit Beclin 1; Alexa Fluor 594 conjugate anti mouse Bcl-2) (1:200) for one hour at room temperature, followed by Hoechst 33342 (0.2 μg/ml) staining for 5 min. After four washes in PBS, cells were mounted with fluorescence mounting medium and examined under confocal microscopes. The objective lens 63X was used for imaging.

### Immunoprecipitation

Co-IP experiments were conducted to show the interaction between GFP-Bcl2 and endogenous Beclin1. PC12 cells were sub-cultured in a 6-well plate and GFP-tagged rat Bcl2 (residues 1–156) plasmids or empty plasmids, in equal amounts, were transfected into PC12 cells using Lipofectamine 3000 (Invitrogen), following the manufacturer’s protocol. MA at the concentration of 10uM was applied to the cells transfected with GFP-tagged rat Bcl2 plasmids or empty plasmids for 12 hours. After treatment, cells were lysed in IP lysis buffer (20 mM HEPES/pH7.4, 1 mM MgCl_2_, 0.25 mM CaCl_2_, 0.1% Triton X-100, 120 mM NaCl, EDTA-free protease inhibitor cocktail (Roche), 200 μg/ml phenylmethylsulfonyl fluoride, pepstatin 4μg/ml, and DNase I). Protein concentrations were measured by Bradford protein assay (Bio-Rad Laboratory, USA). Equal amounts of cytosolic proteins were incubated with GFP antibody overnight at 4°C and then incubated with Dynabeads protein G (Invitrogen) for 1h. The beads were washed in×IP lysis buffer 5 times and then eluted out and analyzed by western blot analysis for the presence of endogenous Beclin1.

### Molecular dynamics

To perform a molecular dynamics (MD) simulation of the binding of MA to Beclin1 or Bcl2, force field parameters of MA were prepared using the Antechamber module in the AMBER software (version14) 36; while Beclin1 or Bcl2 was assigned with FF03SB force field parameters. The complex of Beclin1/Bcl2 and MA was solvated in a TIP3P water box with a margin of 10 Å at each dimension and equilibrated following the standard stepwise using the Sander module in AMBER. Based on the outcomes of MD simulation, the MM-GB/SA method37 implemented in the AMBER software was used to compute the binding affinity of MA to Beclin1/Bcl2. A total of 400 snapshots were sampled from the last 4 ns segment on the entire MD trajectory with an interval of 10 ps. The final binding energy of MA was computed as the average of the results obtained from these 400 snapshots. All parameters used in the MM-GB/SA computation were set to their default values. Vibrational entropy was not considered in our computation.
